# Supplementary material for: High-Resolution Linkage Map and Chromosome-Scale Genome Assembly for Cassava (Manihot esculenta Crantz) from 10 Populations
Source: G3 (Bethesda). 2014 Dec 11;5(1):133–44. doi: 10.1534/g3.114.015008 (PMC4291464; doi:10.1534/g3.114.015008)
Supplement: Supporting Information [file supp_5_1_133__index.html]

High-Resolution Linkage Map and Chromosome-Scale Genome Assembly for Cassava (Manihot esculenta Crantz) from 10 Populations — Supporting Information 

# High-Resolution Linkage Map and Chromosome-Scale Genome Assembly for Cassava (*Manihot esculenta* Crantz) from 10 Populations

## Supporting Information for International Cassava Genetic Map Consortium, 2015

**Files in this Data Supplement:**

- Supporting Information - Figures S1-S4, Tables S1-S2, and Files S1-S2 (PDF, 576 KB)
- Figure S1 - GBS adapter scheme and sequences. (PDF, 108 KB)
- Figure S2 - Singleton markers at the end of LGs. (PDF, 139 KB)
- Figure S3 - Analysis of relatedness in the MT population. (PDF, 116 KB)
- Figure S4 - Merged linkage groups. (PDF, 1 MB)
- Table S1 - Map merging process. (PDF, 103 KB)
- Table S2 - Provenance and pedigrees of parents of mapping populations. (PDF, 137 KB)
- File S1 - Markers excluded from the component maps. (.txt, 6 KB)
- File S2 - Composite genetic map: markers with genetic and physical distances. (.txt, 3 MB)
