# Supplementary material for: High-Resolution Linkage Map and Chromosome-Scale Genome Assembly for Cassava (Manihot esculenta Crantz) from 10 Populations
Source: G3 (Bethesda). 2014 Dec 11;5(1):133–44. doi: 10.1534/g3.114.015008 (PMC4291464; doi:10.1534/g3.114.015008)
Supplement: Supporting Information [file supp_g3.114.015008_015008SI.pdf]

G3· Genes | Genomes | Genetics

High-resolution linkage map and chromosome-scale genome assembly for cassava (*Manihot esculenta* Crantz) from ten populations

International Cassava Genetic Map Consortium (ICGMC)

Corresponding author: Jessica B. Lyons, UC Berkeley Dept. of Molecular and Cell Biology, 541 Life Sciences Addition, Berkeley, CA 94720-3200. E-mail: jblyons@berkeley.edu

Data available in Public Repositories:

- The composite genetic map is available at CassavaBase (http://cassavabase.org/cview/map.pl?map_id=3).
- The pseudomolecule assembly can be downloaded from Phytozome at http://genome.jgi.doe.gov/pages/dynamicOrganismDownload.jsf?organism=Mesculentaunder the directory "v5.1_unreleased".
- Demultiplexed sequence reads, with the barcode and ApeKI cutsite removed, are available in the NCBI Sequence Read Archive via http://www.ncbi.nih.gov/bioproject, Bioproject Accession PRJNA234390.

DOI: 10.1534/g3.114.015008

1. Y-shaped adapters bind DNA via sticky-end ligation

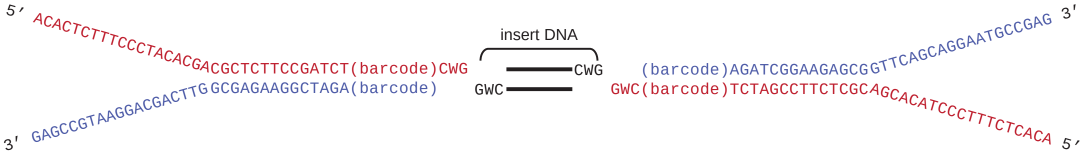

2. PCR

PE1 5′**CAAGCAGAAGACGGCATACGAGATCGGT** CTCGGCATTCCTGCTGAACCGCTCTTCCGATCT binds blue sequence above.
PE2 5′ **AATGATACGGCGACCACCGAGATCT** ACACTCTTTCCCTACACGACGCTCTTCCGATCT binds the complement of red sequence above.

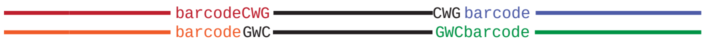

3. Paired-end Sequencing

Paired-end read 1 sequencing primer: 5' ACACTCTTTCCCTACACGACGCTCTTCCGATCT 3' matches red adapter sequence.
Paired-end read 2 sequencing primer: 5' CGGTCTCGGCATTCCTGCTGAACCGCTCTTCCGATCT 3' is complementary to blue adapter sequence.

Figure S1 GBS adapter scheme and sequences. (1) Y-shaped adapters (red/blue) include barcode sequences and a three-base 3’ overhang (CWG) complementary to that left by the ApeKI digestion of genomic DNA (black). The degenerate nucleotide W represents A or T. (2) During PCR amplification, primers PE1 and PE2 add sequences (bold) to the ends of adapter-ligated DNA. These sequences facilitate binding to the flow cell. After the PCR, each double-stranded DNA fragment has a different adapter sequence on each end, and can bind the flow cell. (3) During sequencing, primers bind to DNA strands such that each sequence read begins with the barcode followed by the cut site. Oligonucleotide sequences © 2007-2013 Illumina, Inc. All rights reserved. Derivative works created by Illumina customers are authorized for use with Illumina instruments and products only. All other uses are strictly prohibited.

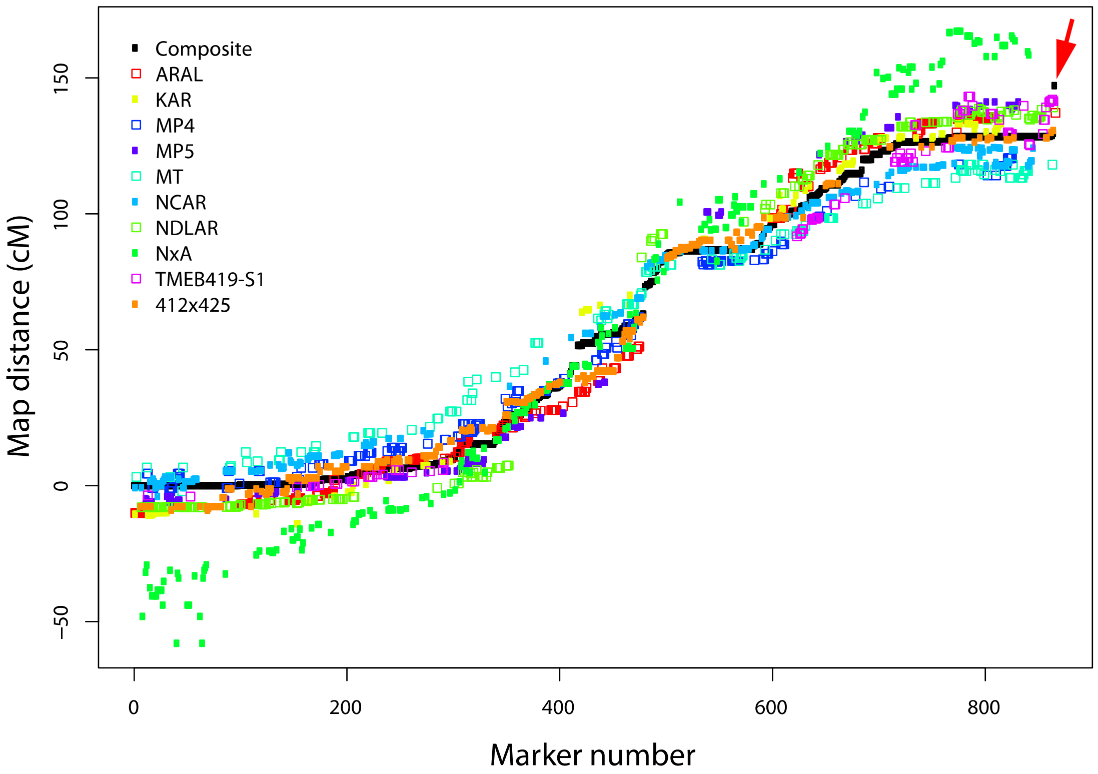

Figure S2 Singleton markers at the end of LGs. The plot shows an example consensus map (Chromosome XII) plotting marker order against genetic distance. The consensus map (filled black squares) contains all markers, and component maps (see legend) each contain a subset. For each component map, an offset was added to the map distance in the plot to minimize the root-mean-squared error (RMSE) distance from the composite map. This helps visualization of large component maps (e.g. NxA) on the same scale. Some markers contributed by a single component map mapped at genetic distance of tens of cM from their neighbors (red arrow) in the merged map. Terminal singleton markers responsible for this behavior were removed and merging was repeated.

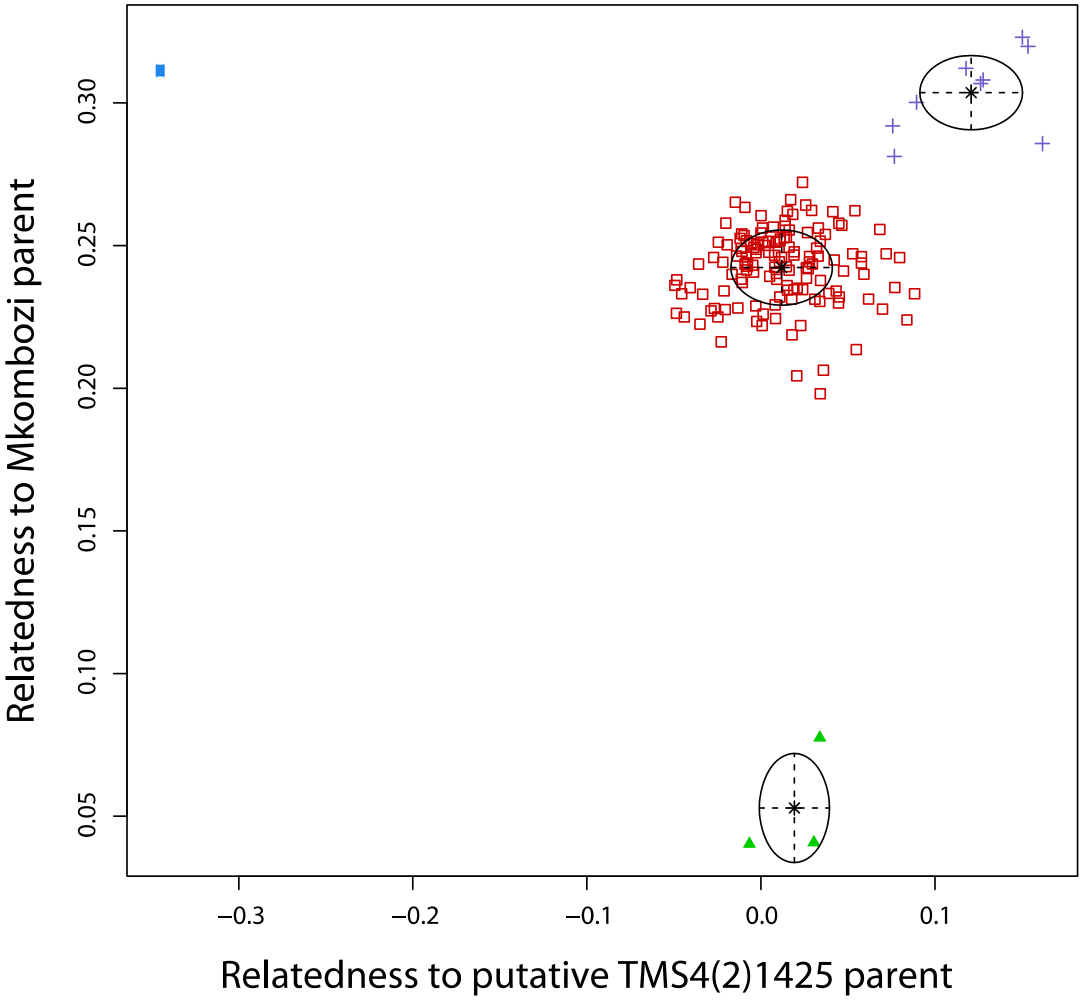

Figure S3 Analysis of relatedness in the MT population. The progeny of the Mkombozi x TMS4(2)1425 cross (MT) are plotted according to their relatedness to the 'TMS4(2)1425' parent and the Mkombozi parent. Note that none of the offspring shows significant relatedness to the 'TMS4(2)1425' parent, hence the inverted commas and label 'putative'. The progeny form four clusters: a single blue square (top left) is consistent with being a self from the Mkombozi parent; the green triangles are unrelated to either parent; the red squares show the expected ~0.25 relatedness to Mkombozi but are unrelated to the 'TMS4(2)1425' parent. Lastly, the relationship of the purple plus signs is unclear and these progeny were excluded from mapping.

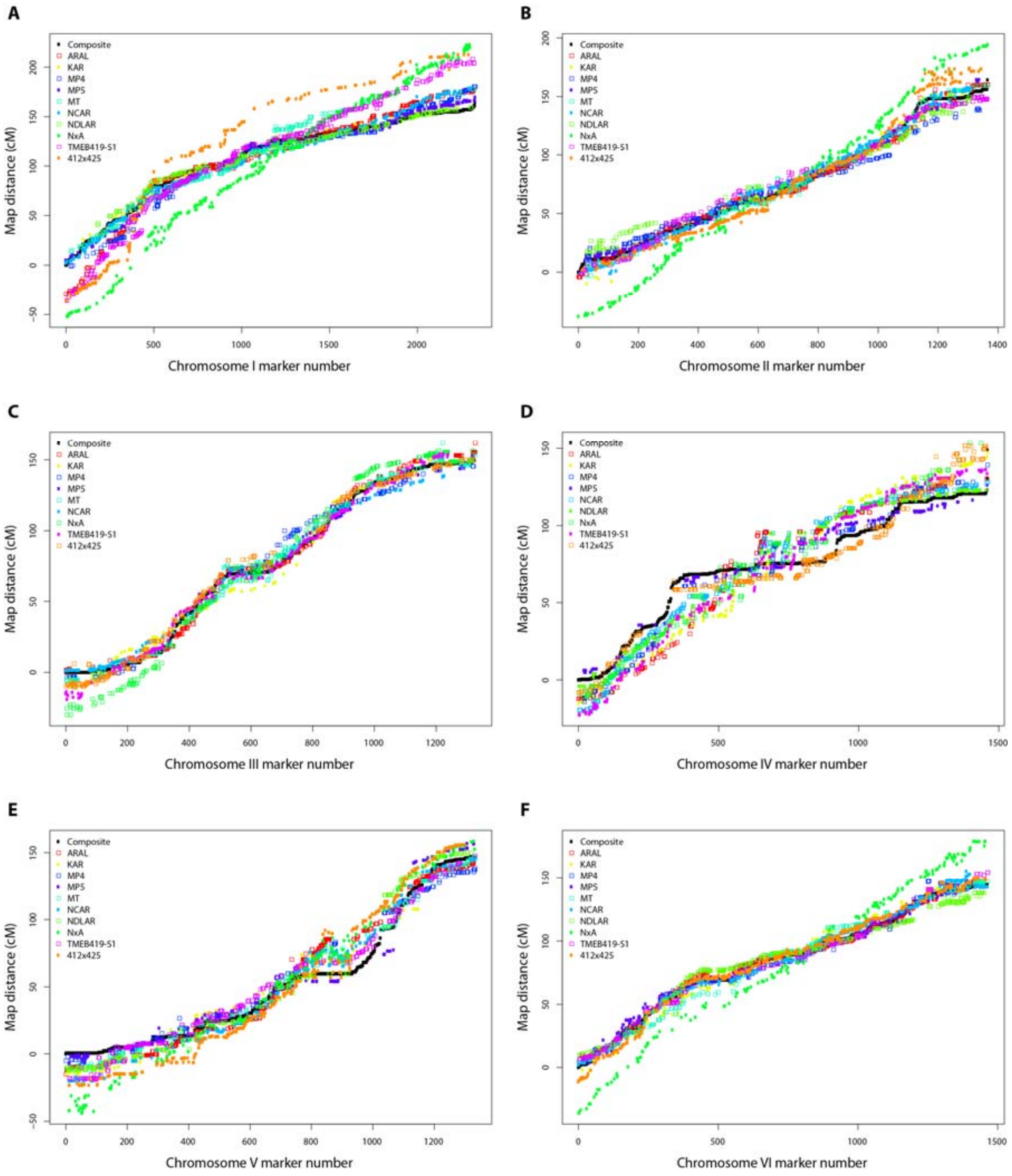
A
Composite
ARAL
KAR
MP4
MP5
MT
NCAR
NDLAR
NxA
TMEB419-S1
412x425
Map distance (cM)
Chromosome I marker number
B
Composite
ARAL
KAR
MP4
MP5
MT
NCAR
NDLAR
NxA
TMEB419-S1
412x425
Map distance (cM)
Chromosome II marker number
C
Composite
ARAL
KAR
MP4
MP5
MT
NCAR
NxA
TMEB419-S1
412x425
Map distance (cM)
Chromosome III marker number
D
Composite
ARAL
KAR
MP4
MP5
NCAR
NDLAR
NxA
TMEB419-S1
412x425
Map distance (cM)
Chromosome IV marker number
E
Composite
ARAL
KAR
MP4
MP5
MT
NCAR
NDLAR
NxA
TMEB419-S1
412x425
Map distance (cM)
Chromosome V marker number
F
Composite
ARAL
KAR
MP4
MP5
MT
NCAR
NDLAR
NxA
TMEB419-S1
412x425
Map distance (cM)
Chromosome VI marker number

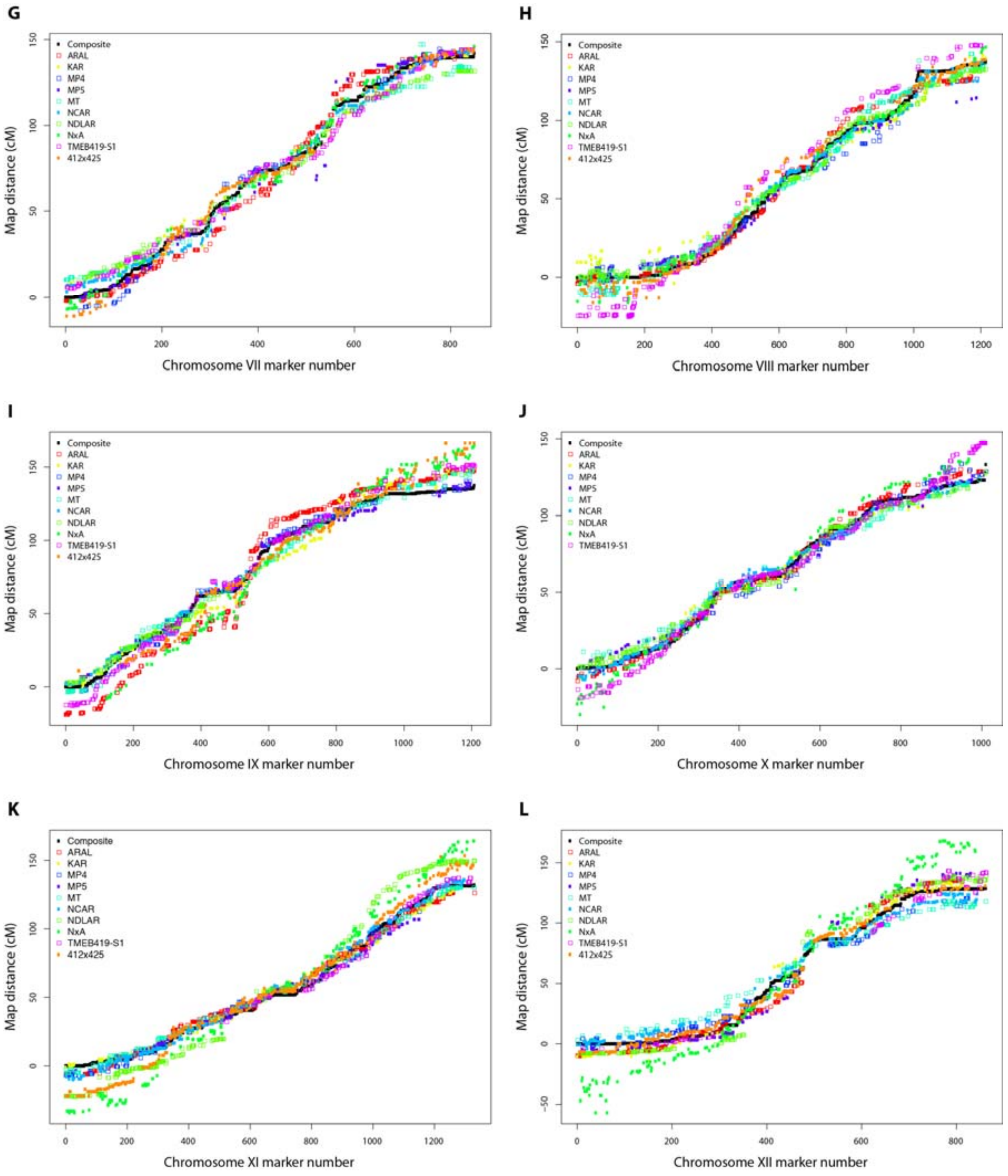
G
H
I
J
K
L
Composite
ARAL
KAR
MP4
MP5
MT
NCAR
NDLAR
NxA
TMEB419-S1
412x425
Map distance (cM)
Chromosome VII marker number
Chromosome VIII marker number
Chromosome IX marker number
Chromosome X marker number
Chromosome XI marker number
Chromosome XII marker number

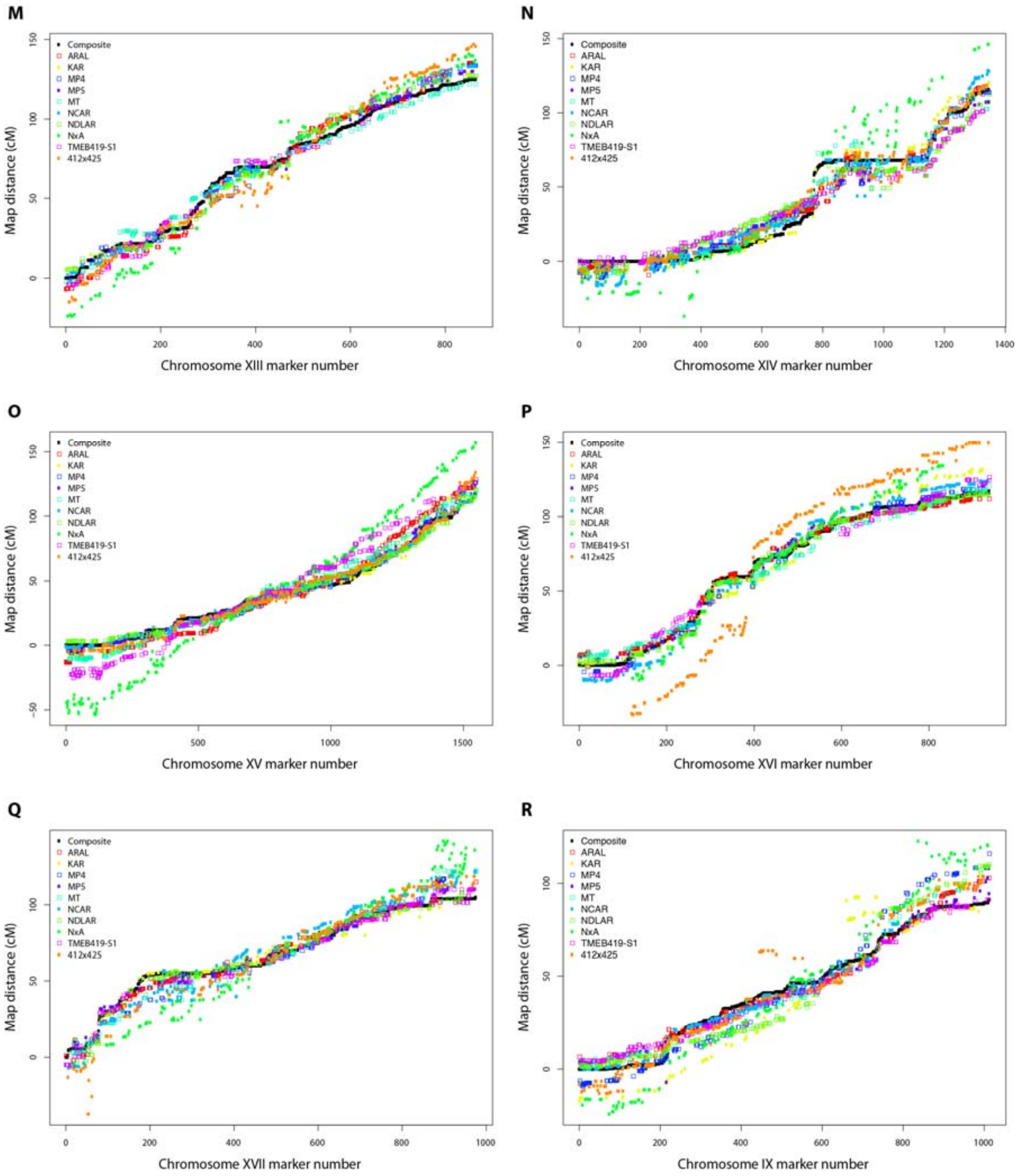

Figure S4 Merged linkage groups. Component maps (colored plot characters) and the merged map (filled black squares) are plotted with marker number along the x-axis against genetic distance. Component maps are shifted in the y-direction to minimize their RMSE relative to the merged map in order to center them by genetic distance.

Table S1 Map merging process. Values of LPmerge parameters and details of the map merging process are shown. See the Materials and Methods section for details. Max int, value of maximum interval that produced the shortest merged map. Rounds merging & filtering, number of rounds of merging and singleton marker filtering required. Total singletons trimmed, total number of singleton markers trimmed from LG ends during rounds of filtering.

Chromosome	ARAL LG number	Max int	Rounds merging & filtering	Total singletons trimmed
I	9	1	2	3
II	7	3	2	10
III	15	2	2	18
IV	5	1	3	9
V	10	1	3	16
VI	11	2	4	15
VII	4	1	2	4
VIII	14	1	3	5
IX	13	1	2	3
X	16	1	2	2
XI	1	1	2	8
XII	8	1	2	3
XIII	18	1	2	16
XIV	3	1	3	33
XV	12	1	2	7
XVI	6	1	2	9
XVII	2	1	4	14
XVIII	17	1	2	7

Table S2 Provenance and pedigrees of parents of mapping populations.

Accession name	Provenance and/or pedigree information
Namikonga	Known as 'Kaleso' in Kenya. Third backcross from inter-specific hybrid (46106/27) from *M. glaziovii* from Amani breeding program (JENNINGS 1960; HILLOCKS AND JENNINGS 2003).
AR40-6	A CIAT cross between a CMD resistant variety C39 from IITA and CW259-42. CW259-42 is a backcross of MTAI 8 (Rayong 60) and an interspecific cross between *M.e.* ssp *flabellifolia* and CM 2766-5.
Kiroba	Farmer variety from Tanzania.
NDL06/132	Breeding line selected at Agricultural Research Institute (ARI) Naliendele in southern Tanzania. It is an S1 of variety NAL90/34 which showed strong resistance to CBSD (HILLOCKS AND JENNINGS 2003) and is half sib of Kibaha, which has *M.e.* ssp. *flabellifolia* background.
Mkombozi	It is a half sib of 92/0099S2(SM). 92/0099 is a cross 91934 x 81/00032. 91934 is 58308 x Ogunjobi, and 81/00032 is a cross between U/1421 and P-2. Also known as MM96/4684. 58308 is from Amani material selected at Moore Estate, and thus has wild species introgression in its pedigree.
Nachinyaya	Farmer variety from Tanzania.
Albert	Farmer variety from Tanzania.
TMS-IBA4(2)1425	58308 x Oyarugba Funfun.
AR37-80	A CIAT cross between a CMD resistant line (C33) from IITA and CW259-42.
TMEB419	TMEB419 is a clone collected in Togo with an unknown pedigree, and released as a variety in Nigeria in 2005 with the name TME419. It is also known by the name Gbasekoute. TMEB419 is very popular all over Nigeria and many other African countries where it has been introduced because it has high dry matter and starch content. The quality of food products from it such as gari and fufu are excellent. It also has erect stems with minimal branding, which facilitates intercropping as well as higher planting densities.
TMS-IBA011412	Cloned in 2001, this is an improved IITA variety with resistance to CMD and it accumulates provitamin A carotenoids in its storage roots. It is the progeny of a cross between TMS-IBA950971 and TMS-IBA940561.
TMS-IBA30001	One of the early TMS series of clones derived from early CMD resistance breeding work in West Africa. It traces its ancestry to variety 58308 which came from interspecific crosses with *M. glaziovii.*
TMS-IBA961089A	TMS-IBA961089A is an improved variety from IITA that shows strong resistance to CMD that is likely to be a result of its parentage. Its female parent is TMS-MOK940461, a half-sib of Nigerian landrace TMEB9, which possesses qualitative resistance to CMD. Its other parent, TMS-IBA9001554, is a half-sib of TMS-IBA30572, an improved variety cloned in 1973, with quantitative resistance to CMD that is derived from variety 58308, a hybrid derived directly from recombination of the *M. glaziovii* × *M. esculenta* triple-backcrosses.

References

Hillocks, R. J., and D. L. Jennings, 2003 Cassava brown streak disease: A review of present knowledge and research needs. Int. J. Pest Manage. 49: 225–234.

Jennings, D. L., 1960 Observations on virus diseases of cassava in resistant and susceptible varieties: I mosaic disease. Empire Journal of Experimental Agriculture 28: 23–34.

File S1-S2

Available for download as .txt files at http://www.g3journal.org/lookup/suppl/doi:10.1534/g3.114.015008/-/DC1

File S1

Markers excluded from the component maps. Markers shown in this table were excluded because they caused inter-marker distances in the individual population maps of 50 cM or greater.

File S2

Composite genetic map: markers with genetic and physical distances. The file contains the following columns: marker ID, chromosome, physical position along the chromosome (bp), genetic position (cM), strand, and approximately 101 bp tags containing all observed alleles (in brackets) with the reference allele always listed first.
