# Supplementary material for: High-Resolution Linkage Map and Chromosome-Scale Genome Assembly for Cassava (Manihot esculenta Crantz) from 10 Populations
Source: G3 (Bethesda). 2014 Dec 11;5(1):133–44. doi: 10.1534/g3.114.015008 (PMC4291464; doi:10.1534/g3.114.015008)
Supplement: Supporting Information [file supp_g3.114.015008_FigureS1.pdf]

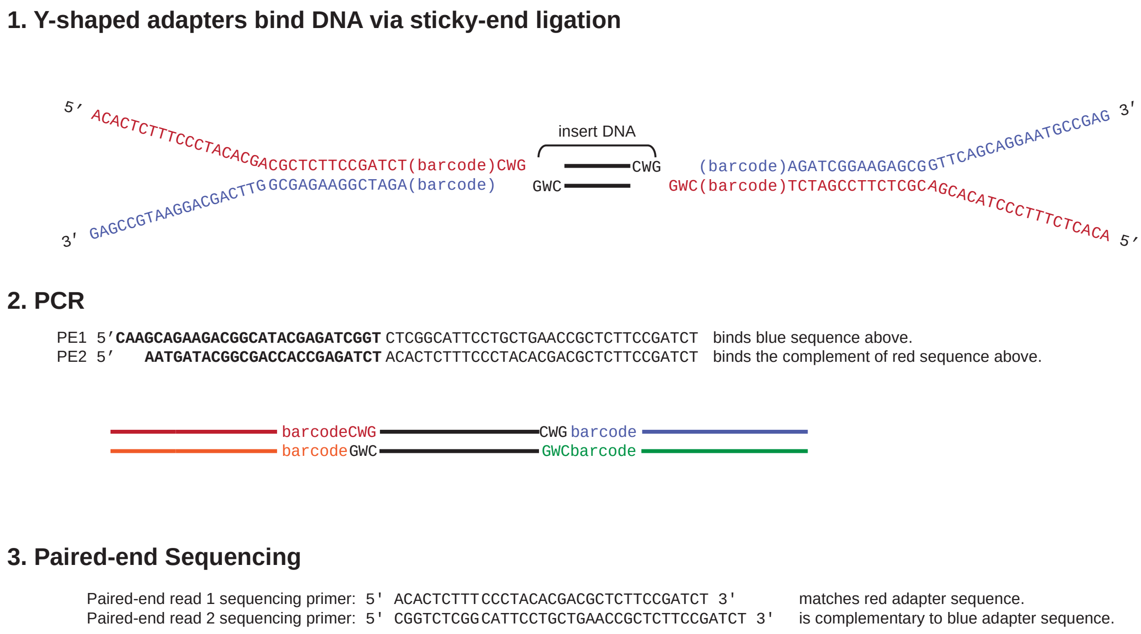

Figure S1 GBS adapter scheme and sequences. (1) Y-shaped adapters (red/blue) include barcode sequences and a three-base 3' overhang (CWG) complementary to that left by the ApeKI digestion of genomic DNA (black). The degenerate nucleotide W represents A or T. (2) During PCR amplification, primers PE1 and PE2 add sequences (bold) to the ends of adapter-ligated DNA. These sequences facilitate binding to the flow cell. After the PCR, each double-stranded DNA fragment has a different adapter sequence on each end, and can bind the flow cell. (3) During sequencing, primers bind to DNA strands such that each sequence read begins with the barcode followed by the cut site. Oligonucleotide sequences © 2007-2013 Illumina, Inc. All rights reserved. Derivative works created by Illumina customers are authorized for use with Illumina instruments and products only. All other uses are strictly prohibited.
