# Supplementary material for: High-Resolution Linkage Map and Chromosome-Scale Genome Assembly for Cassava (Manihot esculenta Crantz) from 10 Populations
Source: G3 (Bethesda). 2014 Dec 11;5(1):133–44. doi: 10.1534/g3.114.015008 (PMC4291464; doi:10.1534/g3.114.015008)
Supplement: Supporting Information [file supp_g3.114.015008_FigureS2.pdf]

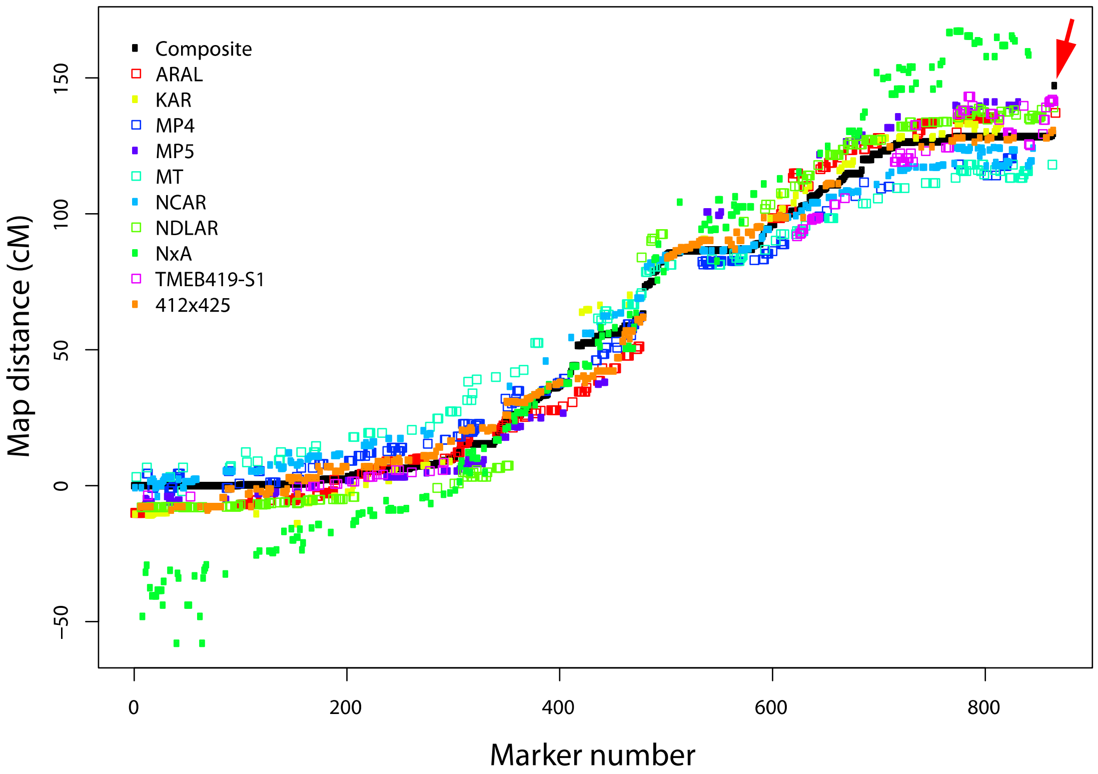

Figure S2 Singleton markers at the end of LGs. The plot shows an example consensus map (Chromosome XII) plotting marker order against genetic distance. The consensus map (filled black squares) contains all markers, and component maps (see legend) each contain a subset. For each component map, an offset was added to the map distance in the plot to minimize the root-mean-squared error (RMSE) distance from the composite map. This helps visualization of large component maps (e.g. NxA) on the same scale. Some markers contributed by a single component map mapped at genetic distance of tens of cM from their neighbors (red arrow) in the merged map. Terminal singleton markers responsible for this behavior were removed and merging was repeated.
