# Supplementary material for: High-Resolution Linkage Map and Chromosome-Scale Genome Assembly for Cassava (Manihot esculenta Crantz) from 10 Populations
Source: G3 (Bethesda). 2014 Dec 11;5(1):133–44. doi: 10.1534/g3.114.015008 (PMC4291464; doi:10.1534/g3.114.015008)
Supplement: Supporting Information [file supp_g3.114.015008_FigureS3.pdf]

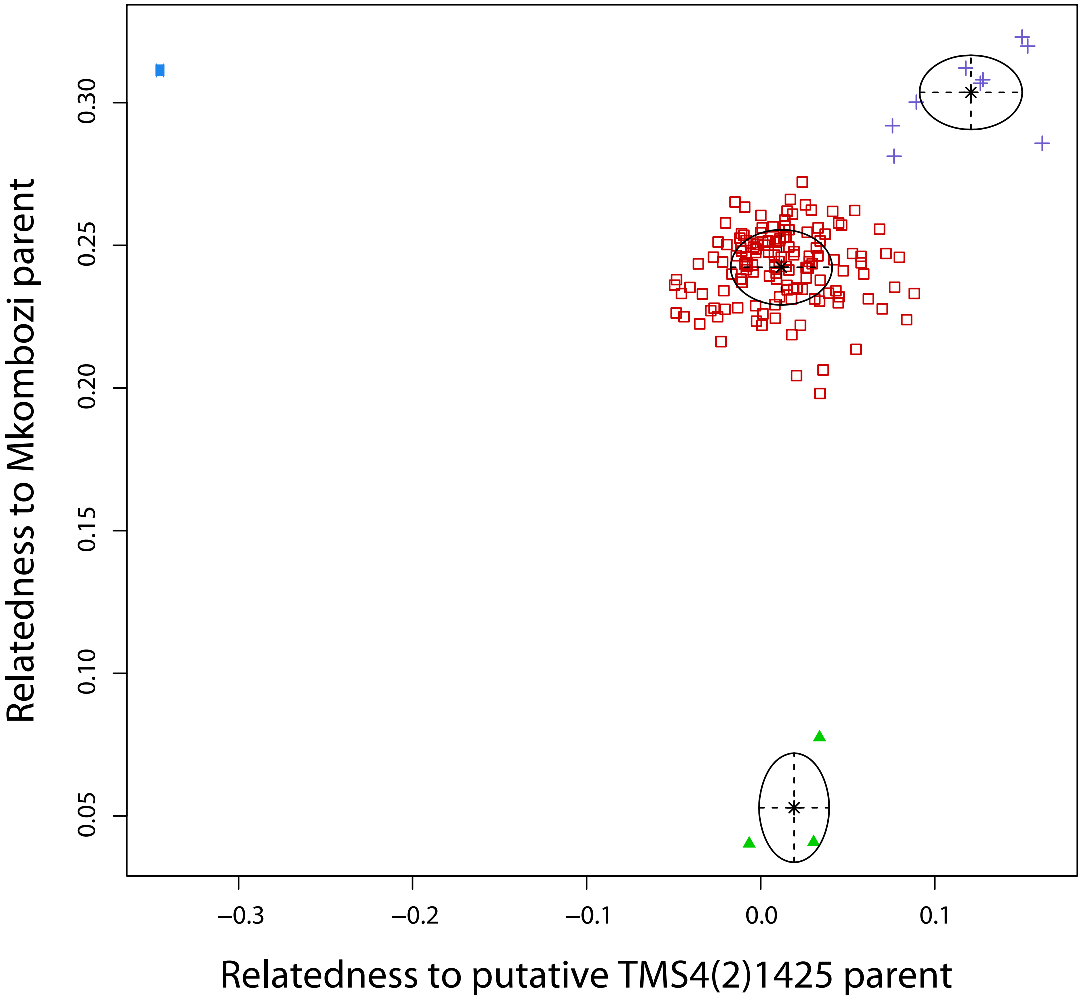

Figure S3 Analysis of relatedness in the MT population. The progeny of the Mkombozi x TMS4(2)1425 cross (MT) are plotted according to their relatedness to the 'TMS4(2)1425' parent and the Mkombozi parent. Note that none of the offspring shows significant relatedness to the 'TMS4(2)1425' parent, hence the inverted commas and label 'putative'. The progeny form four clusters: a single blue square (top left) is consistent with being a self from the Mkombozi parent; the green triangles are unrelated to either parent; the red squares show the expected ~0.25 relatedness to Mkombozi but are unrelated to the 'TMS4(2)1425' parent. Lastly, the relationship of the purple plus signs is unclear and these progeny were excluded from mapping.
