# Supplementary material for: High-Resolution Linkage Map and Chromosome-Scale Genome Assembly for Cassava (Manihot esculenta Crantz) from 10 Populations
Source: G3 (Bethesda). 2014 Dec 11;5(1):133–44. doi: 10.1534/g3.114.015008 (PMC4291464; doi:10.1534/g3.114.015008)
Supplement: Supporting Information [file supp_g3.114.015008_FigureS4.pdf]

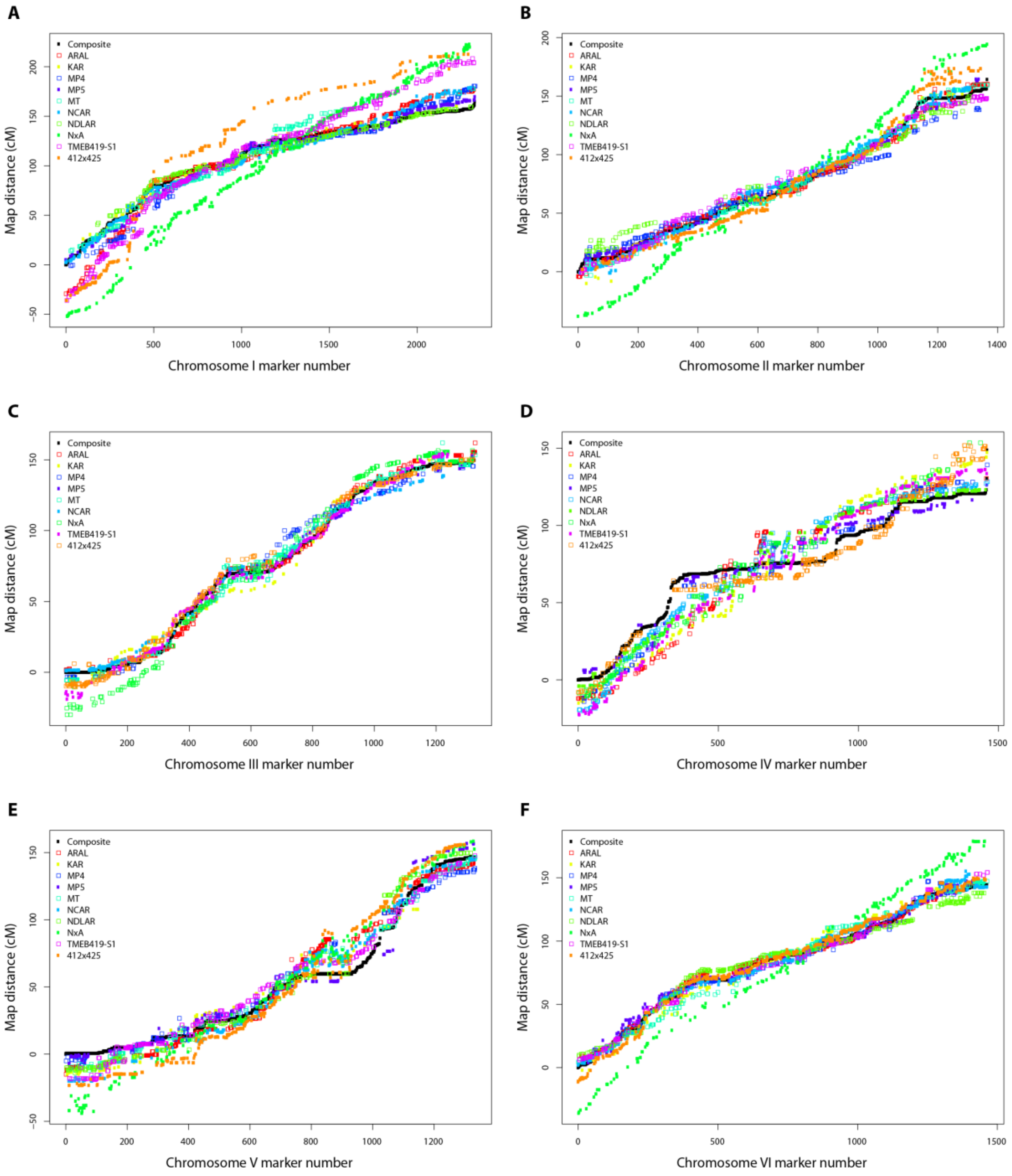
A
Composite
ARAL
KAR
MP4
MP5
MT
NCAR
NDLAR
NxA
TMEB419-S1
412x425
Map distance (cM)
Chromosome I marker number
B
Composite
ARAL
KAR
MP4
MP5
MT
NCAR
NDLAR
NxA
TMEB419-S1
412x425
Map distance (cM)
Chromosome II marker number
C
Composite
ARAL
KAR
MP4
MP5
MT
NCAR
NxA
TMEB419-S1
412x425
Map distance (cM)
Chromosome III marker number
D
Composite
ARAL
KAR
MP4
MP5
NCAR
NDLAR
NxA
TMEB419-S1
412x425
Map distance (cM)
Chromosome IV marker number
E
Composite
ARAL
KAR
MP4
MP5
MT
NCAR
NDLAR
NxA
TMEB419-S1
412x425
Map distance (cM)
Chromosome V marker number
F
Composite
ARAL
KAR
MP4
MP5
MT
NCAR
NDLAR
NxA
TMEB419-S1
412x425
Map distance (cM)
Chromosome VI marker number

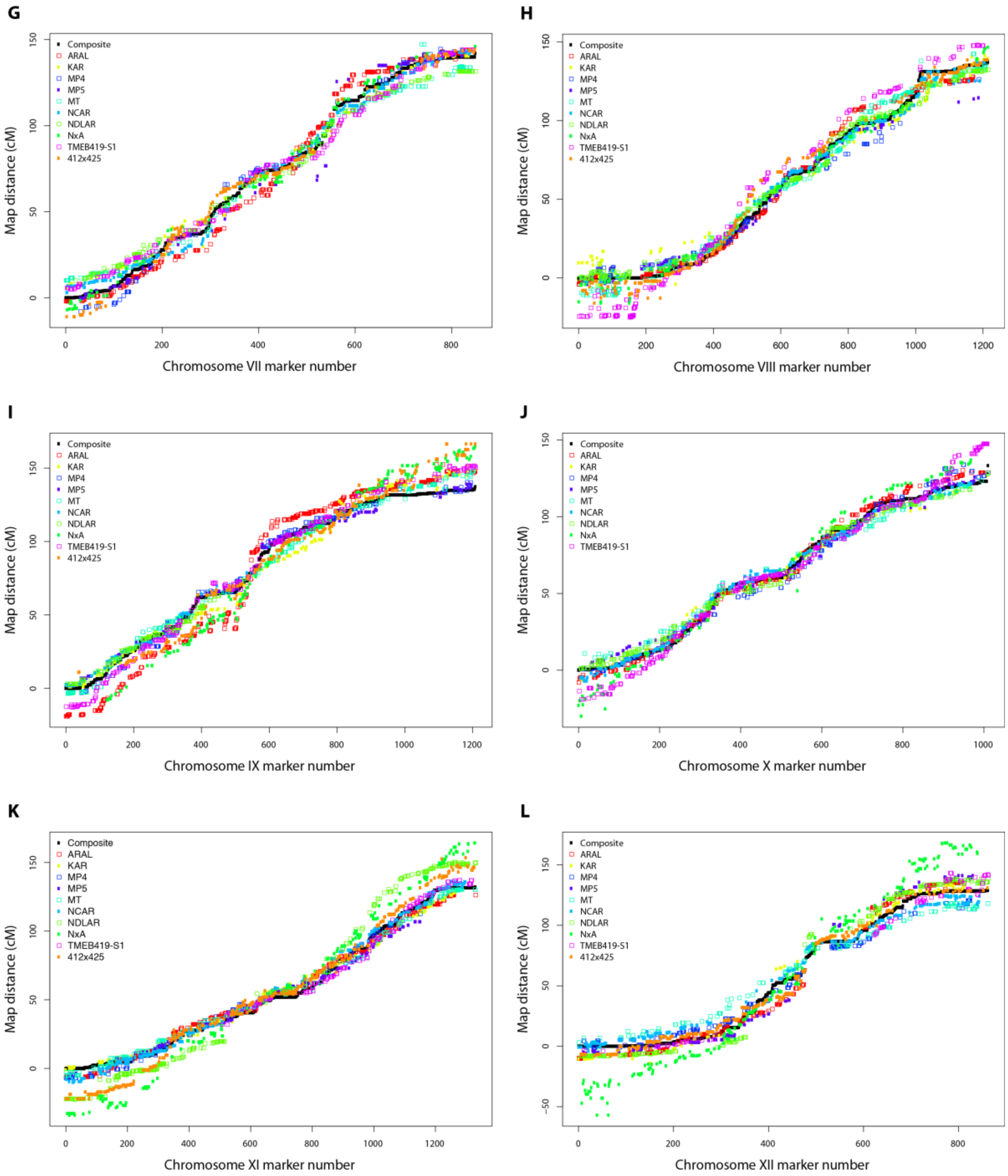
G
Composite
ARAL
KAR
MP4
MP5
MT
NCAR
NDLAR
NxA
TMEB419-S1
412x425
Map distance (cM)
Chromosome VII marker number
H
Composite
ARAL
KAR
MP4
MP5
MT
NCAR
NDLAR
NxA
TMEB419-S1
412x425
Map distance (cM)
Chromosome VIII marker number
I
Composite
ARAL
KAR
MP4
MP5
MT
NCAR
NDLAR
NxA
TMEB419-S1
412x425
Map distance (cM)
Chromosome IX marker number
J
Composite
ARAL
KAR
MP4
MP5
MT
NCAR
NDLAR
NxA
TMEB419-S1
Map distance (cM)
Chromosome X marker number
K
Composite
ARAL
KAR
MP4
MP5
MT
NCAR
NDLAR
NxA
TMEB419-S1
412x425
Map distance (cM)
Chromosome XI marker number
L
Composite
ARAL
KAR
MP4
MP5
MT
NCAR
NDLAR
NxA
TMEB419-S1
412x425
Map distance (cM)
Chromosome XII marker number

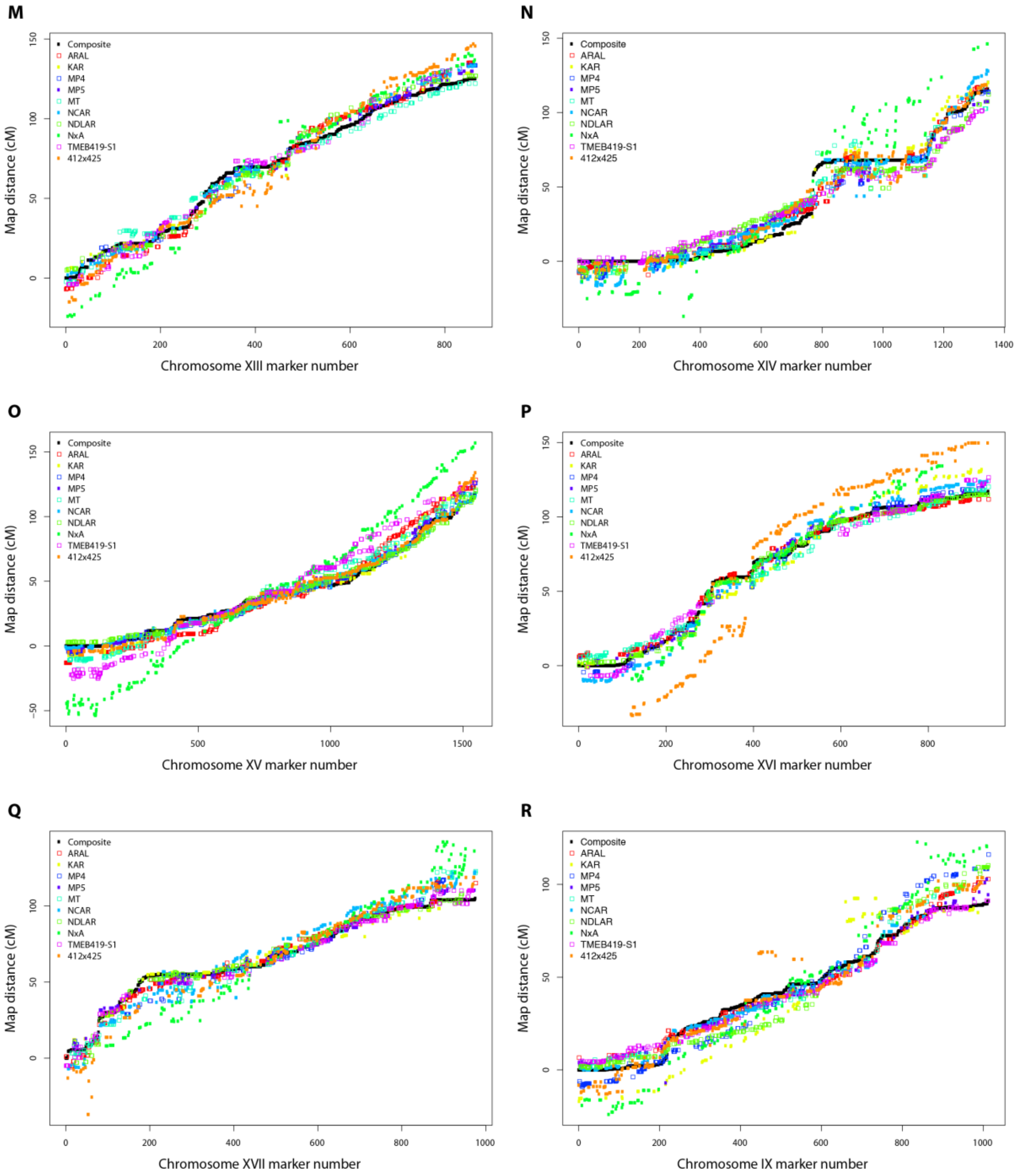

Figure S4 Merged linkage groups. Component maps (colored plot characters) and the merged map (filled black squares) are plotted with marker number along the x-axis against genetic distance. Component maps are shifted in the y-direction to minimize their RMSE relative to the merged map in order to center them by genetic distance.
