# Supplementary material for: High-Resolution Linkage Map and Chromosome-Scale Genome Assembly for Cassava (Manihot esculenta Crantz) from 10 Populations
Source: G3 (Bethesda). 2014 Dec 11;5(1):133–44. doi: 10.1534/g3.114.015008 (PMC4291464; doi:10.1534/g3.114.015008)
Supplement: Supporting Information [file supp_g3.114.015008_TableS1.pdf]

Table S1 Map merging process. Values of LPmerge parameters and details of the map merging process are shown. See the Materials and Methods section for details. Max int, value of maximum interval that produced the shortest merged map. Rounds merging & filtering, number of rounds of merging and singleton marker filtering required. Total singletons trimmed, total number of singleton markers trimmed from LG ends during rounds of filtering.

Chromosome	ARAL LG number	Max int	Rounds merging & filtering	Total singletons trimmed
I	9	1	2	3
II	7	3	2	10
III	15	2	2	18
IV	5	1	3	9
V	10	1	3	16
VI	11	2	4	15
VII	4	1	2	4
VIII	14	1	3	5
IX	13	1	2	3
X	16	1	2	2
XI	1	1	2	8
XII	8	1	2	3
XIII	18	1	2	16
XIV	3	1	3	33
XV	12	1	2	7
XVI	6	1	2	9
XVII	2	1	4	14
XVIII	17	1	2	7
