# Supplementary material for: High-Resolution Linkage Map and Chromosome-Scale Genome Assembly for Cassava (Manihot esculenta Crantz) from 10 Populations
Source: G3 (Bethesda). 2014 Dec 11;5(1):133–44. doi: 10.1534/g3.114.015008 (PMC4291464; doi:10.1534/g3.114.015008)
Supplement: Supporting Information [file supp_g3.114.015008_TableS2.pdf]

Table S2 Provenance and pedigrees of parents of mapping populations.

Accession name	Provenance and/or pedigree information
Namikonga	Known as 'Kaleso' in Kenya. Third backcross from inter-specific hybrid (46106/27) from *M. glaziovii* from Amani breeding program (Jennings 1960; Hillocks and Jennings 2003).
AR40-6	A CIAT cross between a CMD resistant variety C39 from IITA and CW259-42. CW259-42 is a backcross of MTAI 8 (Rayong 60) and an interspecific cross between *M.e.* ssp *flabellifolia* and CM 2766-5.
Kiroba	Farmer variety from Tanzania.
NDL06/132	Breeding line selected at Agricultural Research Institute (ARI) Naliendele in southern Tanzania. It is an S1 of variety NAL90/34 which showed strong resistance to CBSD (Hillocks and Jennings 2003) and is half sib of Kibaha, which has *M.e.* ssp. *flabellifolia* background.
Mkombozi	It is a half sib of 92/0099S2(SM). 92/0099 is a cross 91934 x 81/00032. 91934 is 58308 x Ogunjobi, and 81/00032 is a cross between U/1421 and P-2. Also known as MM96/4684. 58308 is from Amani material selected at Moore Estate, and thus has wild species introgression in its pedigree.
Nachinyaya	Farmer variety from Tanzania.
Albert	Farmer variety from Tanzania.
TMS-IBA4(2)1425	58308 x Oyarugba Funfun.
AR37-80	A CIAT cross between a CMD resistant line (C33) from IITA and CW259-42.
TMEB419	TMEB419 is a clone collected in Togo with an unknown pedigree, and released as a variety in Nigeria in 2005 with the name TME419. It is also known by the name Gbasekoute. TMEB419 is very popular all over Nigeria and many other African countries where it has been introduced because it has high dry matter and starch content. The quality of food products from it such as gari and fufu are excellent. It also has erect stems with minimal branding, which facilitates intercropping as well as higher planting densities.
TMS-IBA011412	Cloned in 2001, this is an improved IITA variety with resistance to CMD and it accumulates provitamin A carotenoids in its storage roots. It is the progeny of a cross between TMS-IBA950971 and TMS-IBA940561.
TMS-IBA30001	One of the early TMS series of clones derived from early CMD resistance breeding work in West Africa. It traces its ancestry to variety 58308 which came from interspecific crosses with *M. glaziovii.*
TMS-IBA961089A	TMS-IBA961089A is an improved variety from IITA that shows strong resistance to CMD that is likely to be a result of its parentage. Its female parent is TMS-MOK940461, a half-sib of Nigerian landrace TMEB9, which possesses qualitative resistance to CMD. Its other parent, TMS-IBA9001554, is a half-sib of TMS-IBA30572, an improved variety cloned in 1973, with quantitative resistance to CMD that is derived from variety 58308, a hybrid derived directly from recombination of the *M. glaziovii* × *M. esculenta* triple-backcrosses.

References

Hillocks, R. J., and D. L. Jennings, 2003 Cassava brown streak disease: A review of present knowledge and research needs. Int. J. Pest Manage. 49: 225–234.

Jennings, D. L., 1960 Observations on virus diseases of cassava in resistant and susceptible varieties: I mosaic disease. Empire Journal of Experimental Agriculture 28: 23–34.
